# Trends in tuberculosis clinicians’ adoption of short-course regimens for latent tuberculosis infection

**DOI:** 10.1016/j.jctube.2023.100382

**Published:** 2023-06-13

**Authors:** Pei-Jean I. Feng, David J. Horne, Jonathan M. Wortham, Dolly J. Katz, for the CDC Tuberculosis Epidemiologic Studies Consortium

**Affiliations:** a Centers for Disease Control and Prevention, 1600 Clifton Rd, Atlanta, GA 30329, USA; b University of Washington School of Medicine and Public Health—Seattle and King County, 3980 15^th^ Avenue NE, Box 351616, Seattle, WA 98195-1616, USA

**Keywords:** Latent tuberculosis infection, Short-course regimens

## Abstract

**Objective:**

Little is known about regimen choice for latent tuberculosis infection in the United States. Since 2011, the Centers for Disease Control and Prevention has recommended shorter regimens—12 weeks of isoniazid and rifapentine or 4 months of rifampin—because they have similar efficacy, better tolerability, and higher treatment completion than 6–9 months of isoniazid. The objective of this analysis is to describe frequencies of latent tuberculosis infection regimens prescribed in the United States and assess changes over time.

**Methods:**

Persons at high risk for latent tuberculosis infection or progression to tuberculosis disease were enrolled into an observational cohort study from September 2012–May 2017, tested for tuberculosis infection, and followed for 24 months. This analysis included those with at least one positive test who started treatment.

**Results:**

Frequencies of latent tuberculosis infection regimens and 95% confidence intervals were calculated overall and by important risk groups. Changes in the frequencies of regimens by quarter were assessed using the Mann-Kendall statistic. Of 20,220 participants, 4,068 had at least one positive test and started treatment: 95% non-U.S.–born, 46% female, 12% <15 years old. Most received 4 months of rifampin (49%), 6–9 months of isoniazid (32%), or 12 weeks of isoniazid and rifapentine (13%). Selection of short-course regimens increased from 55% in 2013 to 81% in late 2016 (p < 0.001).

**Conclusions:**

Our study identified a trend towards adoption of shorter regimens. Future studies should assess the impact of updated treatment guidelines, which have added 3 months of daily isoniazid and rifampin to recommended regimens.

## 1 Background

Reactivation of latent tuberculosis infection (LTBI) accounts for over 80% of tuberculosis (TB) cases in the United States (U.S.) [1]. Because treatment of LTBI can prevent progression to disease, detection and treatment of LTBI is essential to TB elimination (<1.0 case per million persons) in the United States [2].

An estimated 13 million persons in the U.S. have LTBI [3]. Public health departments do not have the capacity to test and treat all persons with LTBI, so engagement of primary care providers of high-risk populations will be essential to achieve TB elimination. Significant reductions in TB rates will also require quadrupling the number of people who complete LTBI treatment, and improving the rate of treatment completion, which ranges from 47% to 82% [2], [4], [5].

Prior to 2000, the recommended LTBI treatment was 12 months of isoniazid. Length of treatment was a major impediment to treatment completion. In 2000, the American Thoracic Society (ATS) and the Centers for Disease Control and Prevention (CDC) recommended four shorter LTBI regimens: 1) 9 months of daily or twice weekly isoniazid (9H), 2) 6 months of daily or twice weekly isoniazid (6H), 3) 2 months of pyrazinamide and rifampin (2RZ), or 4) 4 months of daily rifampin (4R). Of these, 9H was the preferred regimen. In cases where 9H could not be given, 4R and 2RZ were recommended as alternatives [6].

In 2003, CDC recommended against using 2RZ due to high rates of hospitalizations and deaths [7]. In 2011, CDC recommended a new regimen of once-weekly isoniazid and rifapentine for 12 weeks (3HP) given by directly observed therapy (DOT) for HIV-negative persons who are at least 12 years old [8]. In 2018, CDC expanded the 3HP recommendation to include children 2–17 years old and persons with HIV, and be self-administered [9], [10]. In 2020, CDC updated its guidelines by recommending short-course regimens as preferred regimens—3HP, 4R, and 3 months of daily isoniazid and rifampin (3HR)—and 6H or 9H as alternative regimens [11]. Studies have shown that the shorter regimens have similar efficacy and better tolerability and are more likely to be completed than 6H or 9H [12], [13].

Little information has been generated about clinicians’ prescribing practices over time. This information is important to develop guidance and communication tools for health department and community clinicians. To address this knowledge gap, we analyzed data from participants enrolled in a large prospective study of diagnostic tests for LTBI conducted by the TB Epidemiologic Studies Consortium (TBESC), a CDC-funded partnership of 10 academic institutions and public health departments in 11 U.S. states.

The objectives of this sub-analysis were to 1) describe the types and frequencies of LTBI treatment regimens in use at TBESC-affiliated clinics and 2) assess any changes in the types and frequencies of LTBI treatment regimens over time.

## 2 Methods

### 2.1 Study design and patients

Eighteen TBESC-affiliated clinics (Supplementary Table 1) recruited individuals at high risk for LTBI or progression to TB disease from July 2012 through May 2017 to assess the ability of tuberculin skin test (TST) and interferon-gamma release assays (IGRAs) to predict progression to TB disease [14].

A person was eligible for the study if (s)he was (1) a close contact (≥8 h in a week) to an infectious TB case, (2) born in a country whose U.S. population had a high (>100 cases per 100,000 population) rate of TB [15]; (3) a recent arrival (≤5 years) from a country whose U.S. population had a moderate (10–99 cases per 100,000 population) rate of TB [15]; (4) a visitor (≥30 days) in the previous five years to countries whose populations in the U.S. had high rates of TB, (5) living with HIV infection, or (6) members of local populations with documented LTBI prevalence ≥ 25%: homeless persons (two sites) or persons born in countries with moderate rates of TB who had arrived in the U.S. >5 years previously (from Mexico at two sites, from Mexico and El Salvador at one site). Persons were not enrolled if they (1) had a history of anaphylactic reaction to tuberculin; or were (2) already on treatment of TB disease or LTBI, (3) foster children, or (4) planning to leave the United States in <2 years.

At enrollment, each participant had blood drawn for both IGRAs (QuantiFERON-TB Gold In-tube and T-SPOT.*TB*) followed by placement of a TST the same day. For participants with at least one positive test, detailed regimen data, including medications, dosages, frequencies, methods of administration (self-administered or directly observed), and regimen changes, were collected for participants who accepted LTBI treatment. Treatment completion data was collected only for the participant’s latest regimen.

A CDC institutional review board (IRB) reviewed and approved the study protocol, questionnaires, and consent documents. Each TBESC site and its affiliated clinics obtained local IRB approvals or deferred to the CDC IRB. All participants provided written informed consent, assent, or parental/guardian permission. The study is registered at clinicaltrials.gov (NCT01622140).

### 2.2 Treatment regimens

For purposes of this analysis, we included only participants who 1) had at least one positive test result, 2) were offered LTBI treatment, and 3) accepted LTBI treatment. We categorized 4R and 3HP as short-course regimens, and 6H and 9H as long-course.

We categorized all other regimens as nonstandard.

### 2.3 Statistical analysis

For participants who received LTBI treatment, we calculated descriptive statistics for participants’ demographics and their risk factors for LTBI or progression to TB disease. We calculated the frequencies of LTBI treatment regimens and compared these frequencies by risk groups with risk ratios (RR) and 95% CI.

We calculated frequencies of LTBI treatment regimens by quarter from January 2013 through September 2016 to visualize changes in LTBI regimen prescription patterns over time for all clinics combined and for each clinic with at least 20 participants who received LTBI treatment. We calculated the Mann-Kendall statistic to detect any significant increase or decrease in the prescription of short-course regimens over time.

We also calculated treatment completion proportions stratified by treatment regimen and duration. The definition of “treatment completion” was established by each site, which may be based upon number and frequency of doses and/or the provider’s judgment. For those who did not complete the initially prescribed regimen, we summarized the reasons for not completing treatment by frequency and proportion. Since we do not have the reason for not completing the initial treatment regimen for participants who changed regimens during the study, we categorized the reason for incomplete treatment for these participants as “unknown.”.

## 3 Results

### 3.1 Study population

**Fig. 1:**
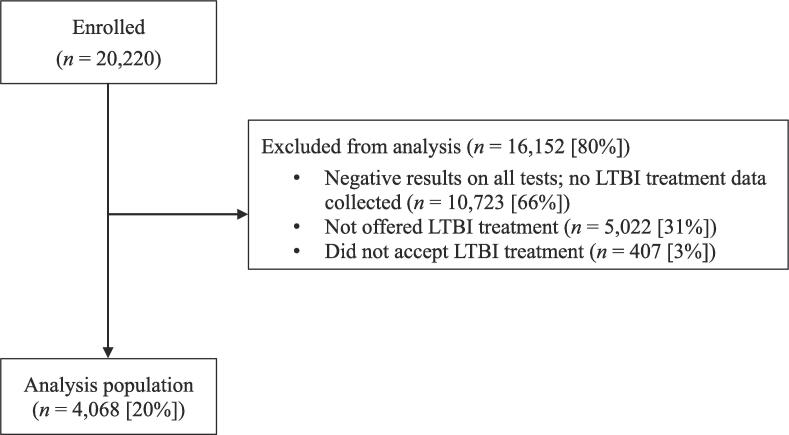
Latent tuberculosis infection treatment regimen study flow diagram, 18 U.S. clinics, 2012–2017.

Of the 20,220 participants enrolled in the main study, 16,152 participants were excluded from this analysis for a variety of reasons: 66% (10,723/16,152) lacked at least one positive test result, 31% (5,022/16,152) were not offered LTBI treatment, and 3% (407/16,152) did not accept treatment. The 4,068 included in the analysis had at least one positive TST or IGRA, were offered LTBI treatment, and accepted treatment (Fig. 1).

Almost half (1,854/4,068) of the 4,068 were female, 56 (3%) of whom were pregnant; >70% (2,929/4,068) were at least 25 years old (median age: 34), and almost all (95%, 3,859/4,068) were non-U.S.–born. Besides birth outside the United States, other risk factors for LTBI or progression to TB included close contact to an infectious TB case (14%, 561/4,068), and HIV infection (2%, 83/4,068) (Table 1).

**Table 1:** Demographic, medical, and social risk characteristics of participants diagnosed with latent tuberculosis infection who accepted treatment, 18 U.S. clinics, 2012–2017.

Characteristic	*n* (%)
N	4,068 (1 0 0)
Gender^a^	
Male	2,212 (54)
Female	1,854 (46)
Age, mean (IQR)	34 (24, 46)
Age group (Years)	
<15	468 (12)
15–24	671 (16)
25–44	1,865 (46)
45–64	916 (23)
≥65	148 (4)
Race or ethnicity^b^^,^^c^	
Asian	1,592 (39)
Black or African American	787 (19)
White or Caucasian	160 (4)
Hispanic or Latino	388 (10)
Other	1,004 (25)
Risk factors for TB or LTBI^b^	
Close contact	561 (14)
Non-U.S.–born	3,859 (95)
Class B1 immigrant	523 (13)
Self-report HIV-positive	83 (2)
Liver disease	69 (2)
Chronic kidney failure	14 (0)
Diabetes	181 (4)
Immunosuppressive therapy	46 (1)

TB = tuberculosis, LTBI = latent tuberculosis infection.

a Two participants reported being transgender.

b Not mutually exclusive.

c Race or ethnicity is unknown for or not answered by 182 participants.

### 3.2 Treatment regimens

The three most commonly prescribed LTBI regimens were 4R (49%, 1,999/4,068), 6/9H (32%, 1,303/4,068) and 3HP (13%, 542/4,068) (Table 2). All participants who were prescribed 4R and almost all (93%, 1,215/1,303) of those who were prescribed 6/9H, received the treatment via self-administered therapy (SAT). Ninety-six percent (518/542) of those prescribed 3HP had treatment administered via directly observed therapy, as recommended by CDC during the time of the study. Most children < 15 years old received 6/9H, as did participants with HIV infection.

**Table 2:** Treatment regimens for latent tuberculosis infection by age, race or ethnicity and risk factors among participants who accepted treatment, 18 U.S. clinics, 2012–2017.

Characteristic	4R^a^ *n* (%)	6/9H^b^ *n* (%)	3HP^c^ *n* (%)
N (*n* = 4,068)	1,999 (49)	1,303 (32)	542 (13)
Age group (Years)			
<15 (*n* = 468)	134 (29)	314 (67)	14 (3)
15–24 (*n* = 671)	356 (53)	211 (31)	83 (12)
25–44 (*n* = 1,864)	1,007 (54)	457 (25)	324 (17)
45–64 (*n* = 917)	434 (47)	282 (31)	105 (12)
≥65 (*n* = 148)	68 (46)	37 (25)	16 (11)
Race or ethnicity^d^^,^^e^			
Asian (*n* = 1,592)	932 (59)	394 (25)	152 (10)
Black or African American (*n* = 786)	300 (38)	371 (47)	104 (13)
White or Caucasian (*n* = 160)	54 (34)	74 (46)	29 (18)
Hispanic or Latino (*n* = 389)	152 (39)	180 (47)	40 (10)
Risk factors for TB or LTBI^d^			
Close contact (*n* = 562)	214 (38)	235 (42)	92 (16)
Non-U.S.–born (*n* = 3,861)	1,968 (51)	1,175 (30)	498 (13)
Class B1 immigrant (*n* = 523)	230 (44)	91 (17)	51 (10)
Self-report HIV-positive (*n* = 83)	3 (4)	75 (90)	0 (0)

Note: all percentages are row percentages. The denominators are written as (n = #).

TB = tuberculosis, LTBI = latent tuberculosis infection.

a 4 months rifampin, daily.

b 6 or 9 months isoniazid, daily.

c 12 weeks of isoniazid and rifapentine, weekly.

d Not mutually exclusive.

e Race or ethnicity is unknown for or not answered by 182 participants.

Six percent (224/4,068) of participants were prescribed nonstandard regimens, which included 4 months of isoniazid and rifampin (4HR) (61%, 137/224), empiric treatment of TB disease with isoniazid, rifampin, pyrazinamide, and ethambutol (RIPE) (26%, 58/224); rifamycin-containing regimens where rifampin was substituted with rifabutin (4%, 9/224); and fluoroquinolones (7%, 15/224). Treatment with RIPE is usually considered to represent complete LTBI treatment after eight weeks in persons determined not to have TB disease [11]. Over 70% (141/195) of participants who received either 4HR or RIPE were recent immigrants whose overseas radiographs were read as abnormal, but whose overseas laboratory tests for TB were negative. Such persons, designated Class B1 immigrants, are at higher risk of TB disease [16], [17], [18]. All participants prescribed a fluoroquinolone-containing regimen were close contacts to persons with multidrug-resistant TB disease.

The types of standard regimens prescribed varied across clinics. One clinic prescribed 4R only, five prescribed 4R and 6/9H, and 12 prescribed 4R, 6/9H, and 3HP. Of the five clinics that prescribed both 4R and 6/9H, three prescribed 6/9H to at least 85% of their participants. The other two clinics in this group favored 4R over 6/9H. Among the 12 clinics that prescribed all standard regimens, nine prescribed 4R most often; two, 6/9H; and one, 3HP (Supplemental Table 1).

The type of treatment regimen prescribed differed by age. About two-thirds (67%, 316/468) of children <15 years old were given 6/9H, compared to 27% (987/3,600) of participants who were at least 15 years old (RR = 2.1, 95% CI = 1.9–2.4). For all ages, Black or African American, White, or Hispanic or Latino participants were most commonly prescribed 6/9H (46%–47%), followed by 4R (34%–39%) and 3HP (10%–18%). Among Asian participants, 59% (932/1,592), 25% (394/1,592) and 10% (152/1,592) were prescribed 4R, 6/9H and 3HP, respectively (Table 2). The most common regimen prescribed to both non-U.S.–born participants (51%, 1,968/3,861) and Class B1 immigrants (44%, 230/523) was 4R, while close contacts were most often prescribed 6/9H (42%, 235/562). These categories are not mutually exclusive.

### 3.3 Treatment prescription trends

**Fig. 2:**
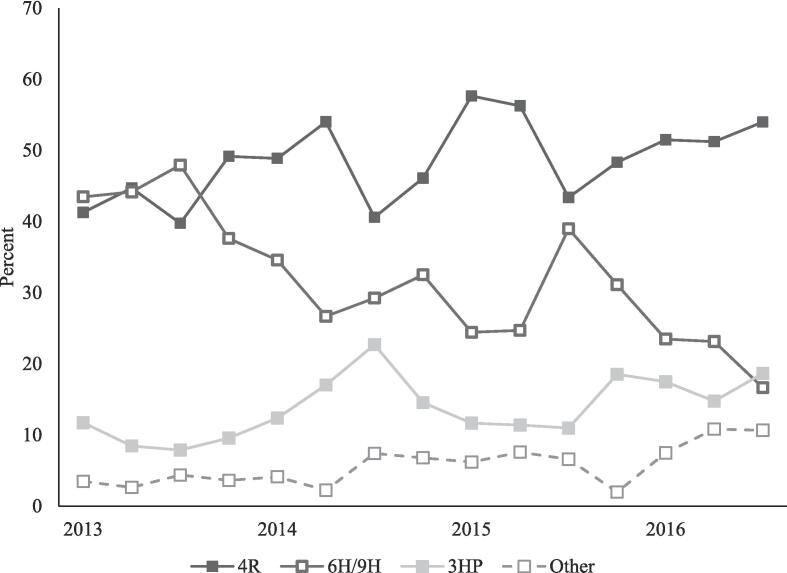
Percentage of latent tuberculosis infection treatment regimens^a^ prescribed, by quarter, 18 U.S. clinics, 2013–2016. ^a^ 4R: 4 months of rifampin, daily; 6/9H: 6 or 9 months of isoniazid, daily; 3HP: 12 weeks of isoniazid and rifapentine, weekly.

**Fig. 3:**
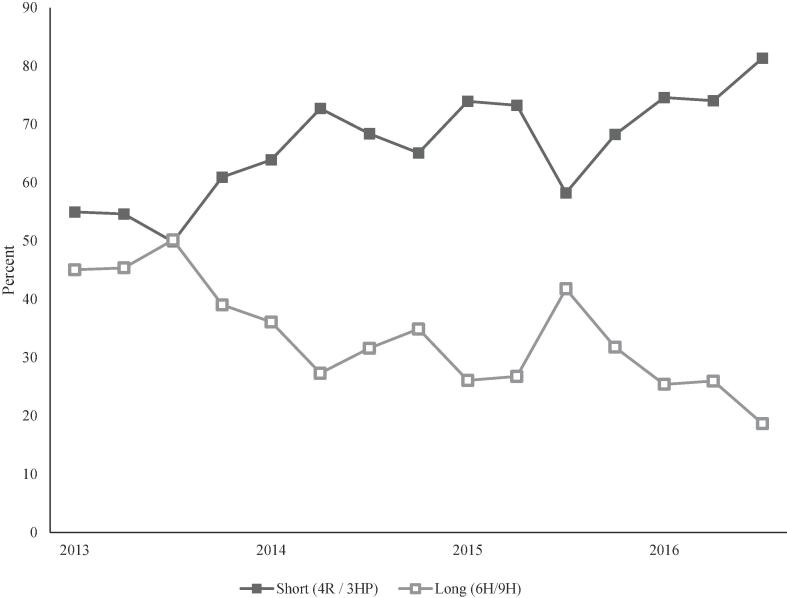
Percentage of short vs long latent tuberculosis infection treatment regimens^a^ prescribed, by quarter, 18 U.S. clinics, 2013–2016. ^a^ 4R: 4 months of rifampin, daily; 6/9H: 6 or 9 months of isoniazid, daily; 3HP: 12 weeks of isoniazid and rifapentine, weekly.

The two most commonly prescribed regimens in 2013 were 4R and 6/9H, each around 40% of all regimens prescribed. From January 2013 to September 2016, the percent of participants who were prescribed 1) 4R climbed from 41% (95/230) to 54% (81/150), 2) 6/9H decreased from 44% (100/230) to 17% (25/150), and 3) 3HP increased from 12% (27/230) in 2013 to 19% (28/150) in 2016 (Fig. 2). Among those prescribed a standard regimen, the percentage of short-course regimens prescribed over time increased from 55% (122/222) to 81% (109/134) (Kendall’s τ = 0.1, p < 0.001), with a clear separation favoring the short-course regimens seen starting in September 2013 (Fig. 3). The difference between the long and short-course regimens continued to grow through September of 2016.

Trends in selection of treatment regimens varied among clinics and displayed three patterns: an upward or downward trend in the use of short-course regimens or a consistent use of short-course regimens over time. Some clinics did not have enough data to detect a pattern. Three clinics showed small decreases in the use of short-course regimens over time—Baltimore City Health Department (Kendall’s τ = − 0.3, p = 0.004), Seattle-King County Public Health (Kendall’s τ = − 0.2, p = 0.001), and Maricopa County Public Health Department (Kendall’s τ = − 0.2, p-value < 0.001). A significant increase in the use of short-course regimens was seen at the Florida Department of Health—Fort Lauderdale (Kendall’s τ = 0.2, p = 0.001), the County of San Diego Health and Human Services Agency (Kendall’s τ = 0.4, p < 0.001), and the Tarrant County Health Department (Kendall’s τ = 0.7, p < 0.001). Some clinics consistently used short-course regimens throughout the study—Atrium Health, DeKalb County Board of Health, Denver Public Health, Hawaii Department of Health, and San Francisco Department of Public Health. One clinic continued to prescribe 6/9H as its predominant regimen—Florida Department of Health—Broward County. The remaining five clinics had too few observations to detect any pattern—University of California San Diego Antiviral Research Center, Florida Department of Health—Gainesville, Wake County Health Department, Montgomery County Health Department and Florida Department of Health—Miami-Dade County. The overall trend in increased use of short-course regimens was driven primarily by the three clinics that saw an increase in the usage of short-course regimens, which accounted for 26% of patients.

### 3.4 Treatment completion

Treatment completion was 82% (2,089/2,541) for participants prescribed a short-course regimen and 67% (873/1,303) for participants prescribed 6/9H regimens (Supplemental Table 2). The most common reasons for not completing treatment for both the short-course (41%) and the long-course regimen (49%) was lost to follow-up (Supplemental Table 3).

## 4 Discussion

In this study, we found that the frequency of short-course rifampin-based regimens increased over the study period compared to 6/9H. We observed that regimen use varied by site, participant age, ethnicity, and risk factors. Children and participants with HIV infection tended to receive 6/9H, which was the recommended regimen for these populations at the time of the study. Treatment completion was greater among participants prescribed a short-course regimen than those prescribed a long-term regimen.

As stated earlier, CDC’s LTBI treatment guidelines have evolved over time. In 2000, shorter regimens were included as alternatives to 6/9H. As more evidence of the safety, efficacy, and adherence of short-course regimens became available over time, CDC gradually changed its recommendations, preferring short-course regimens over 6/9H, which is now considered alternative treatment. Our study adds to the body of evidence that persons on shorter course regimens are more likely to complete treatment.

Our analysis, which included LTBI treatment prescription data through 2017, predates 2018 and 2020 updates in LTBI treatment guidelines. Therefore, we were unable to assess the impact of 3HP SAT and 3HR. However, with the trend toward shorter regimens that we see in our study, use of 3HP, one of the shortest regimens and with the least number of doses, is likely to increase, especially with the removal of the DOT requirement and the expansion of the recommendation to include children at least 2 years old and persons with HIV [11].

With a trend toward shorter regimens, TBESC clinics still strongly favored 4R rather than the shortest regimen, 3HP. This suggests that factors other than duration of treatment are taken into account when establishing standard LTBI treatment practices at a clinic, such as potential drug-drug interactions, adverse events, the medication costs, and pill burden [11], [12]. 3HP requires 10 pills to be taken simultaneously once weekly compared to two or three pills daily for 4R and 6/9H. Another consideration is a clinician’s familiarity with using both rifampin and rifapentine. Clinicians unfamiliar with these drugs may inadvertently confuse the two [11]. Other possible explanations for variation in choice of LTBI treatment regimen include availability and cost of staff to administer DOT treatment, and the local TB disease incidence [11]. Finally, specific patient characteristics may dictate a specific choice: two clinics deviated from their primary regimen of 4R for Class B1 immigrants and prescribed 4HR instead, which is consistent with ATS guidelines for treatment of persons with abnormalities on their chest radiographs after TB disease has been ruled out [6].

Our data on adoption of new LTBI treatments is consistent with research on other new medicines. Studies show that new treatments have a curve of adoption; some clinicians are rapid adopters, while others follow along more slowly, depending on a variety of physician and patient factors [19], [20]. Complex regimens may take 10 years or more to be adopted by a majority of clinicians [21]. Current guidelines for LTBI diagnosis and treatment are complex and leave room for interpretation on the best approach to testing and treatment [22]. Moreover, continuing improvements in treatment regimens have resulted in periodic changes in CDC recommendations; one could argue that the clock of acceptance resets each time a new change is recommended. With more changes likely as new regimens continue to be tested and introduced, we should expect the trend towards prescription of shorter regimens to be irregular, but upward.

Limitations of our study include that we did not collect data about factors that influence prescribing practices, so could not assess clinic and clinician factors that contributed to the wide variations between sites. In addition, we did not collect the duration of the isoniazid regimen offered, so we were unable to distinguish between 6 months and 9 months of isoniazid. Lastly, our study captured only the prescribing patterns of TB experts in public health and academic settings. We do not know if the same patterns apply to providers in primary care and community health settings. Since engagement of community providers is essential for TB elimination [2] information needs to be gathered on how LTBI treatment is prescribed in the community. As an effort is made to expand LTBI testing and treatment in communities with persons at high risk for LTBI or progression to TB disease, the prescribing practices of community providers will greatly affect if and when we reach TB elimination.

## 5 Conclusions

Our study identified a trend towards adoption of shorter regimens for treatment of individuals at high risk of LTBI or progression to TB disease, with 4R the most popular regimen. Future studies are needed to understand the full impact of the updated LTBI treatment guidelines, which have added 3HR and 3HP self-administered therapy to the list of recommended regimens.

## Funding

This work was funded by the Centers for Disease Control and Prevention.

Consent

All participants provided written informed consent, assent, or parental/guardian permission.

## CRediT authorship contribution statement

**Pei-Jean I. Feng:** Methodology, Software, Formal analysis, Writing – original draft, Visualization. **David J. Horne:** Writing – review & editing. **Jonathan M. Wortham:** Writing – review & editing. **Dolly J. Katz:** Conceptualization, Methodology, Supervision.

## Declaration of Competing Interest

The authors declare that they have no known competing financial interests or personal relationships that could have appeared to influence the work reported in this paper.
