# Supplementary material for: Trends in tuberculosis clinicians’ adoption of short-course regimens for latent tuberculosis infection
Source: J Clin Tuberc Other Mycobact Dis. 2023 Jun 13;33:100382. doi: 10.1016/j.jctube.2023.100382 (PMC10320582; doi:10.1016/j.jctube.2023.100382)
Supplement: Supplementary data 1 [file mmc1.docx]

**Supplemental Table 1. LTBI treatment regimens prescribed, by U.S. clinic site, 2012–2017**

| **Site** | **4R^a^**  ***n* (%)** | **6/9H^b^**  ***n* (%)** | **3HP^c^**  ***n* (%)** |
| --- | --- | --- | --- |
| Dekalb County Board of Health (*n* = 883) | 649 (74) | 122 (14) | 51 (6) |
| Tarrant County Health Department (*n* = 554) | 40 (7) | 247 (45) | 259 (47) |
| Maricopa County Department of Public Health (*n* = 440) | 254 (58) | 179 (41) | 4 (1) |
| Atrium Health (*n* = 368) | 286 (78) | 73 (20) | 4 (1) |
| Public Health - Seattle and King County (*n* = 337) | 162 (48) | 55 (16) | 115 (34) |
| Denver Public Health (*n* = 327) | 281 (86) | 16 (5) | 17 (5) |
| Florida Department of Health/Broward County—Ft. Lauderdale (*n* = 238) | 19 (8) | 201 (85) | 0 (0) |
| San Francisco Department of Public Health (*n* = 203) | 77 (38) | 62 (31) | 37 (18) |
| Hawaii Department of Health (*n* = 181) | 106 (59) | 16 (9) | 9 (5) |
| Metro Public Health Department—Nashville (*n* = 171) | 2 (1) | 127 (74) | 41 (24) |
| Florida Department of Health/Broward County—Pompano Beach (*n* = 129) | 5 (4) | 121 (94) | 0 (0) |
| County of San Diego Health and Human Services Agency (*n* = 97) | 32 (33) | 34 (35) | 1 (1) |
| Baltimore City Health Department (*n* = 89) | 58 (65) | 29 (33) | 2 (2) |
| Florida Department of Health/Alachua County—Gainesville (*n* = 21) | 13 (62) | 8 (38) | 0 (0) |
| Wake County Health Department (*n* = 10) | 5 (50) | 4 (40) | 1 (10) |
| University of California San Diego Antiviral Research Center (*n* = 8) | 1 (13) | 7 (88) | 0 (0) |
| Florida Department of Health/Miami-Dade County—Miami (*n* = 7) | 4 (57) | 2 (29) | 1 (13) |
| Montgomery County Health Department (*n* = 5) | 5 (100) | 0 (0) | 0 (0) |

^a^ 4 months rifampin, daily

^b^ 6 or 9 months isoniazid, daily

^c^ 12 weeks of isoniazid and rifapentine, weekly

Note: all percentages are row percentages. The denominators are written as (n = #).

LTBI = latent tuberculosis infection

**Supplemental Table 2. Treatment completion by duration of LTBI treatment regimen prescribed, 2012–2017**

| **Treatment duration** | **Completed treatment**  ***n* (%)** | | **Did not complete treatment**  ***n* (%)** | | **Completion status unknown**  ***n* (%)** | | **Total who accepted treatment** |
| --- | --- | --- | --- | --- | --- | --- | --- |
| Short^a^ | 2089 | (82.2) | 447 | (17.6) | 5 | (0.2) | 2541 |
| Long^b^ | 873 | (67.0) | 429 | (32.9) | 1 | (0.1) | 1303 |
| Other | 183 | (81.7) | 40 | (17.9) | 1 | (0.4) | 224 |

^a^ 4 months rifampin, daily or 12 weeks of isoniazid and rifapentine, weekly

^b^ 6 or 9 months isoniazid, daily

Note: all percentages are row percentages.

LTBI = latent tuberculosis infection

**Supplemental Table 3. Reasons for incomplete treatment by duration of LTBI treatment regimen prescribed, 2012–2017**

| **Treatment duration** | **Did not complete treatment**  ***n* (%)** | | **Reason treatment regimen prescribed was not completed** | | | | | | | | | | | | | | | | | |
| --- | --- | --- | --- | --- | --- | --- | --- | --- | --- | --- | --- | --- | --- | --- | --- | --- | --- | --- | --- | --- |
|  |  |  | **Lost to follow-up**  ***n* (%)** | | **Refused**  ***n* (%)** | | **Moved**  ***n* (%)** | | **Diagnosed with TB disease**  ***n* (%)** | | **Side effects**  ***n* (%)** | | **Pregnant**  ***n* (%)** | | **Died**  ***n* (%)** | | **Other**  ***n* (%)** | | **Unknown**  ***n* (%)** | |
| Short^a^ | 447 | (17.6) | 183 | (40.9) | 71 | (15.9) | 72 | (16.1) | 1 | (0.2) | 52 | (11.6) | 16 | (3.6) | 2 | (0.4) | 21 | (4.7) | 29 | (6.5) |
| Long^b^ | 429 | (32.9) | 209 | (48.7) | 59 | (13.8) | 63 | (14.7) | 2 | (0.5) | 32 | (7.5) | 17 | (4.0) | 1 | (0.2) | 23 | (5.4) | 23 | (5.4) |
| Other | 40 | (17.9) | 14 | (35.0) | 8 | (20.0) | 3 | (7.5) | 3 | (7.5) | 9 | (22.5) | 1 | (2.5) | 0 | (0.0) | 1 | (2.5) | 1 | (2.5) |

^a^ 4 months rifampin, daily or 12 weeks of isoniazid and rifapentine, weekly

^b^ 6 or 9 months isoniazid, daily

Note: all percentages are row percentages.

LTBI = latent tuberculosis infection
